# Sequential Interferon β-Cisplatin Treatment Enhances the Surface Exposure of Calreticulin in Cancer Cells via an Interferon Regulatory Factor 1-Dependent Manner

**DOI:** 10.3390/biom10040643

**Published:** 2020-04-21

**Authors:** Pei-Ming Yang, Yao-Yu Hsieh, Jia-Ling Du, Shih-Chieh Yen, Chien-Fu Hung

**Affiliations:** 1 Department of Pathology, Johns Hopkins Medical Institutions, Baltimore, MD 21231, USA; b00401082@ntu.edu.tw; 2 Graduate Institute of Cancer Biology and Drug Discovery, College of Medical Science and Technology, Taipei Medical University, Taipei 11031, Taiwan; small.lyn@gmail.com; 3 PhD Program for Cancer Molecular Biology and Drug Discovery, College of Medical Science and Technology, Taipei Medical University and Academia Sinica, Taipei 11031, Taiwan; 4 TMU Research Center of Cancer Translational Medicine, Taipei 11031, Taiwan; 5 Cancer Center, Wan Fang Hospital, Taipei Medical University, Taipei 11696, Taiwan; 6 Division of Hematology and Oncology, Taipei Medical University Shuang Ho Hospital, New Taipei City 23561, Taiwan; alecto39@gmail.com; 7 Division of Hematology and Oncology, Department of Internal Medicine, School of Medicine, College of Medicine, Taipei Medical University, Taipei 11031, Taiwan; 8 National Taiwan University College of Medicine, Taipei 10051, Taiwan; 9 Department of Oncology, Johns Hopkins Medical Institutions, Baltimore, MD 21231, USA

**Keywords:** bioinformatics, chemotherapy, endoplasmic reticulum stress, immunogenic cell death, interferon

## Abstract

Immunogenic cell death (ICD) refers to a unique form of cell death that activates an adaptive immune response against dead-cell-associated antigens. Accumulating evidence indicates that the efficacy of conventional anticancer agents relies on not only their direct cytostatic/cytotoxic effects but also the activation of antitumor ICD. Common anticancer ICD inducers include certain chemotherapeutic agents (such as anthracyclines, oxaliplatin, and bortezomib), radiotherapy, photodynamic therapy (PDT), and oncolytic virotherapies. However, most chemotherapeutic reagents are inefficient or fail to trigger ICD. Therefore, better understanding on the molecular determinants of chemotherapy-induced ICD will help in the development of more efficient combinational anticancer strategies through converting non- or relatively weak ICD inducers into bona fide ICD inducers. In this study, we found that sequential, but not concurrent, treatment of cancer cells with interferon β (IFNβ), a type I IFN, and cisplatin (an inefficient ICD inducer) can enhance the expression of ICD biomarkers in cancer cells, including surface translocation of an endoplasmic reticulum (ER) chaperone, calreticulin (CRT), and phosphorylation of the eukaryotic translation initiation factor alpha (eIF2α). These results suggest that exogenous IFNβ may activate molecular determinants that convert cisplatin into an ICD inducer. Further bioinformatics and in vitro experimental analyses found that interferon regulatory factor 1 (IRF1) acted as an essential mediator of surface CRT exposure by sequential IFNβ-cisplatin combination. Our findings not only help to design more effective combinational anticancer therapy using IFNβ and cisplatin, but also provide a novel insight into the role of IRF1 in connecting the type I IFN responses and ICD.

## 1. Introduction

Accumulating evidence indicates that the efficacies of conventional anticancer agents rely on not only their direct cytostatic/cytotoxic effects but also the activation of tumor-targeting immune responses [1]. One such response is immunogenic cell death (ICD), indicating that dying cancer cells can elicit an effective antitumor immune response through the release of damage-associated molecular patterns (DAMPs). DAMPs are recognized by antigen-presenting cells (APCs) such as dendritic cells (DCs), which subsequently activate tumor antigen-specific T cell responses [2,3]. ICD was initially discovered through the findings that tumor xenografts in syngeneic immunocompetent mice, when compared to those in immunodeficient hosts., are more efficiently killed by chemotherapy with anthracyclines (such as mitoxantrone and doxorubicin) or oxaliplatin [4,5]. In addition, cancer cells exposed to a lethal dose of ICD inducers in vitro and then inoculated into syngeneic immunocompetent mice in the absence of adjuvants efficiently protect the animals from a subsequent challenge with living cancer cells of the same type [6]. The original concept of ICD from earlier studies was restricted to classical apoptosis [4,5,7,8,9,10]. However, accumulating evidence suggests that dying cancer cells through other forms of cell death, such as autophagy, necroptosis, and pyroptosis, can also induce ICD [11,12,13]. Given that tumors are often resistant to certain death pathways, the elasticity of multimodal ICD induction may overcome the anticancer drug resistance.

The release of DAMPs are the hallmark and effectors of ICD. The majority of DAMPs during ICD display one of the following actions: (1) cell surface exposure of endoplasmic reticulum (ER) chaperones such as calreticulin (CRT), ERp57, and heat-shock proteins 70/90 kDa (HSP70/90) [7,10,14]; (2) autophagy-mediated secretion of adenosine triphosphate (ATP) [8,11]; and (3) release of the chromatin-binding protein high-mobility group B1 (HMGB1) and annexin A1 (ANXA1) [15,16]. These DAMPs may function as “eat me” and “find me” signals for professional phagocytes through interacting with different phagocytic or scavenger receptors including low density lipoprotein receptor-related protein 1 (LRP1)/cluster of differentiation 91 (CD91) for CRT, purinergic receptors for ATP, pattern-recognition receptors such as Toll-like receptor 4 (TLR4) for HMGB1, and formyl peptide receptor (FPR1) for ANXA1. In addition, to prime an adaptive immune response, ICD is associated with the establishment of immunological memory that has the potential to eradicate malignant cells that survive chemotherapy via an IFNγ-dependent mechanism [2]. Therefore, it is believed that these immunological side effects are helpful and desirable for cancer therapy, and a better understanding of their regulation will facilitate the design of novel combinatorial regimens with improved clinical efficacy.

ICD can be induced by certain chemotherapeutic agents (such as anthracyclines, oxaliplatin, and bortezomib), radiotherapy, photodynamic therapy (PDT), and oncolytic virotherapies [1]. However, most chemotherapeutic agents are inefficient or fail to trigger ICD [4,5,9], which is due to their intrinsic inability to promote the release of one or more of the DAMPs (or the activation of the underlying stress responses). For example, in contrast with its derivative oxaliplatin, cisplatin cannot efficiently trigger the expression of ICD biomarkers [17,18]. It is proposed that the effect of cisplatin on ICD depends on cell type, the concentration used, or treatment duration [19], which suggests that the potential of a chemotherapeutic agent could be manipulated. Indeed, combinational treatment with ER stress inducers (such as thapsigargin or tunicamycin), type I IFNs (co-administration of IFNα and IFNβ), zinc, or vitamin B6 precursor pyridoxine has been found to convert cisplatin into an efficient ICD inducer [17,20,21,22].

Currently, ICD induction by a given agent cannot be predicted. Even the molecules with similar structures or chemical properties do not share the same functional profile to induce ICD. Therefore, a better understanding on the molecular determinants of chemotherapy-induced ICD will help in the development of combinational strategies to convert non- and relatively weak ICD inducers into bona fide ICD inducers. Our results identified that sequential combination of IFNβ and cisplatin was more potent to induce the surface CRT exposure compared to a concurrent combination protocol. Bioinformatics analyses and in vitro experimental validation indicated that IFNβ/IRF1 signaling might be required for the conversion of cisplatin to a more efficient ICD inducer.

## 2. Materials and Methods

### 2.1. Chemicals and Reagents

Roswell Park Memorial Institute-1640 (RPMI-1640) medium (22400071), L-glutamine (25030081), sodium pyruvate (11360070), and antibiotic-antimycotic solution (penicillin G, streptomycin, and amphotericin B; 15240062), fetal bovine serum (FBS; 10437028), Lipofectamine RNAiMAX transfection reagent (13778150), and M-PER mammalian protein extraction reagent (78505) were purchased from ThermoFisher Scientific (San Jose, CA, USA). The 7-amino-actinomycin D (7-AAD; 420404) was purchased from BioLegend (San Diego, CA, USA). Bio-Rad Protein Assay (5000006) and precast sodium dodecyl sulfate (SDS)-polyacrylamide gels (4561023, 4561043, and 4561083) were purchased from Bio-Rad Laboratories (Hercules, CA, USA). Phospho-eIF2α (#9721), IRF1 (#8478), and JUN (#9165) antibodies were purchased from Cell Signaling Technology (Beverly, MA, USA). The eIF2α (sc-11386) antibody was purchased from Santa Cruz Biotechnology (Santa Cruz, CA, USA). The phycoerythrin (PE)-conjugated calreticulin (CRT; ab209577) antibody was purchased from Abcam (Cambridge, MA, USA). β-Actin (GTX109639) and GAPDH (GTX100118) antibodies were purchased from GeneTex (Hsinchu, Taiwan). Horseradish peroxidase (HRP)-labeled secondary antibodies (111-035-003, 115-035-003, and 705-035-003) were purchased from Jackson ImmunoResearch Laboratories (West Grove, PA, USA). The enhanced chemiluminescence (ECL) system (PK-NEL105) was purchased from Perkin-Elmer (Boston, MA, USA). The cis-Diammineplatinum(II) dichloride (cisplatin; P4394), 3-(4,5-Dimethyl-2-thiazolyl)-2,5-diphenyl-2H-tetrazolium bromide (MTT; M2128), and dimethyl sulfoxide (DMSO; D2650 for cell stock storage and D5979 for drug preparation) were purchased from Sigma (St. Louis, MO, USA). The recombinant human interferon β (IFNβ; 300-02BC) was purchased from Peprotech (London, UK). ON-TARGETplus JUN siRNA (L-003268-00-0005) and non-targeting siRNA pool (D-001810-10-05) were purchased from Dharmacon (Lafayette, CO, USA). Blood/Cell DNA Mini Kit (GB100) and GenepHlow Gel/PCR Kit (DFH100) were purchased from Geneaid Biotech (New Taipei City, Taiwan). The OneTaq 2X Master Mix with Standard Buffer (M0482S) was purchased from New England Biolabs (Beverly, MA, USA).

### 2.2. Cell Culture

The human cervical adenocarcinoma HeLa, human cervical squamous cell carcinoma SiHa, and human ovarian adenocarcinoma SKOV3 cells were purchased from American Type Culture Collection (ATCC, Rockville, MD, USA). The mouse lung carcinoma TC-1 [23] cells were provided by Professor T.-C. Wu (Department of Pathology, Johns Hopkins Medical Institutions, Baltimore, MD, USA). The mouse ovarian surface epithelial cells (MOSEC) ovarian cancer cells were prepared as described previously [24]. Cells were cultured at 37 °C in RPMI-1640 medium supplemented with 10% FBS, 1% L-glutamine, 1 mM sodium pyruvate, and 1% antibiotic-antimycotic solution and incubated in a humidified incubator containing 5% CO^2^.

### 2.3. Treatments

For concurrent treatment (cotreatment) of IFNβ and cisplatin, cells were treated simultaneously with IFNβ and/or cisplatin for the indicated time intervals. For sequential treatment of IFNβ and cisplatin, cells were treated first with or without IFNβ for 24~72 h and then washed twice with PBS to remove any residual IFNβ. Cells were subsequently treated with cisplatin (cisplatin or IFNβ+cisplatin) or left untreated (control or IFNβ) for 4 h (Western blotting for protein expression) and 24 h (flow cytometry for surface CRT staining). For the experiments of 72-h MTT cell viability assays, IFNβ-treated cells were washed, trypsinized, and replated in 96-well plates. One day later, cells were treated with cisplatin for 72 h.

### 2.4. Establishment of Interferon Regulatory Factor 1 (IRF1)-Knockout Cells

IRF1-knockout HeLa cells were generated using the clustered, regularly interspaced palindromic repeats (CRISPR)/CRISPR-associated protein 9 (Cas9) system, which was performed by ToolGen (Seoul, South Korea). The sequence of single-guide (sg)RNA used to target the human IRF1 gene was CTCGGATGCGCATGAGACCCTGG. The underlined sequence is the protospacer adjacent motif (PAM) that can be recognized by the Cas9 protein. After transfection with CRISPR/Cas9 plasmids, cells were serially diluted into 96-well plates for clonal expansion. Two independent clones from different wells were chosen for further experiments. For the validation of the sgRNA target site, genomic DNA was extracted and PCR was performed using the following primer pair: GGGTGCCCTACCTCAAG AAG (forward) and AAAGAAGTCCCTCCCTTCCC (reverse). The PCR products were purified and then sequenced by the Sanger method using the forward and reverse primers. The results indicated that a 52-bp deletion was generated in two IRF1-knockout clones (Figure S1).

### 2.5. siRNA Knockdown Analysis

Cells were transiently transfected with human JUN siRNA and non-targeting siRNA using the Lipofectamine RNAiMAX transfection reagent according to the manufacturer’s instruction. The transfection mixture was replaced with fresh, regular medium 24 h later and cells were used for further experiments.

### 2.6. Flow Cytometry

The antibody (ab209577 from Abcam, Cambridge, MA, USA) for surface CRT staining was chosen according to previous studies [25]. Cells were detached by trypsinization and washed once by phosphate-buffered saline (PBS)/1% FBS buffer. Freshly dispersed cell suspension (1 x 10^6^ cells/mL) was stained in PE-conjugated anti-CRT (1:5000) antibody-containing PBS/1% FBS buffer for 30 min on ice in the dark. Then, cells were washed twice by PBS/1% FBS and resuspended in 7-AAD-containing PBS to label the dead cells. The fluorescence was analyzed on the FACSCalibur flow cytometry system (BD Biosciences, San Jose, CA, USA) or the Muse Cell Analyzer (Millipore, Bedford, MA, USA). The results were analyzed using FCSalyzer-0.9.18-alpha (https://sourceforge.net/projects/fcsalyzer/) and 7-AAD-negative cells were gated to exclude dead cells. For the generation of ecto-CRT histograms, only the attached cells were collected and a total of 10,000 7-AAD-negative cells were acquired for analysis. For the generation of ecto-CRT/7-AAD dot plots, both attached and floating cells were collected and a total of 10,000 cells were acquired for analysis.

### 2.7. Cell Viability Assay

Cells were plated in 96-well plates and treated with drugs. Four hours before cell harvest, 0.5 mg/mL of MTT was directly added to each well. The blue MTT formazan precipitate was then dissolved in 200 μL of DMSO. The absorbance at 550 nm was measured on a microplate reader (BioTek, Winooski, VT, USA).

### 2.8. Western Blot Analysis

Cells were lysed with the M-PER mammalian protein extraction reagent on ice for 30 min. Cell lysates were then centrifuged at 13,000× *g* for 20 min at 4 °C. Supernatant was collected and the protein concentration was determined by the Bio-Rad Protein Assay. Equal amounts of protein (50 μg) are resolved in 7.5–13% precast SDS-polyacrylamide gel and then transferred to a nitrocellulose membrane. The membrane was incubated with the appropriate primary antibody at 4 °C overnight. Then, the membrane was washed and incubated with a horseradish peroxidase-conjugated secondary antibody for 30 min at room temperature. The immunoblots were visualized by ECL reagent.

### 2.9. Bioinformatics Analysis of Public Data

The microarray data sets for cisplatin- and oxaliplatin-treated A2780 human ovarian cancer cells (GSE8057 [26]) and cisplatin- and doxorubicin-treated HeLa human cervical cancer cells (GSE72905 [27] and GSE30988 [28]) were obtained from the Gene Expression Omnibus (GEO) database at the National Center for Biotechnology Information (NCBI) [29]. Gene set enrichment analysis (GSEA v4.0.3 software (Broad institute, Cambridge, MA, USA) was used to analyze these data sets for the enrichment of 50 cancer hallmarks [30,31,32]. Genes were ranked by running a gene set type permutation test with Log_2_ ratio ranking statistics. Default settings were used for other parameters. For the visualization of overlap hallmarks or genes, the Venn diagrams were generated using the VENNY 2.1 web tool (https://bioinfogp.cnb.csic.es/tools/venny/). Pathway construction was performed using the STRING (Search Tool for the Retrieval of Interacting Genes/Proteins; http://string-db.org/) database [33]. The parameters were set as follows: organism = homo sapiens; meaning of network edges = molecular action; active interaction source = experiments and databases; minimum required interaction score = high confidence (0.700); max number of interactors to show = none; and network display mode = interactive svg.

### 2.10. Statistical Analysis

Means and standard deviations of samples were calculated from the numerical data with at least three replicates. Survival curves were fit using nonlinear regression. Data were analyzed using Student’s *t*-test, and *p* values of <0.05 were considered statistically significant. Other statistical analyses were performed by the built-in programs in each database used in this study.

## 3. Results

### 3.1. Sequential Interferon β (IFNβ) and Cisplatin Treatment Enhances the Surface Calreticulin (CRT) Exposure in Cancer Cells

**Figure 1:**
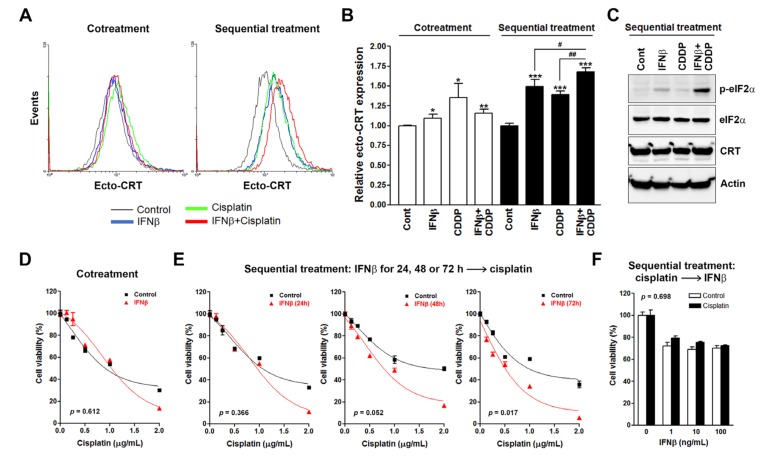
Effects of interferon β (IFNβ) and cisplatin treatment protocols on cell viability and surface calreticulin expression in HeLa cells. (**A**) HeLa cells were cotreated with 100 ng/mL IFNβ and 2 μg/mL cisplatin for 24 h, or sequentially treated with 100 ng/mL IFNβ for 24 h and 2 μg/mL cisplatin for another 24 h. Surface calreticulin (CRT) (ecto-CRT) staining was performed and analyzed by flow cytometry. (**B**) The mean fluorescence of ecto-CRT in (**A**) was quantified and plotted. *p* < 0.05 (* or ^#^), *p* < 0.01 (** or ^##^) and *p* < 0.001 (***) indicate significant differences compared to control samples or the indicated group. (**C**) HeLa cells were sequentially treated with 100 ng/mL IFNβ for 24 h and 2 μg/mL cisplatin for another 4 h. Protein expression was analyzed by Western blotting. (**D**–**F**) For the concurrent treatment (cotreatment) protocol (**D**), HeLa cells were treated with 100 ng/mL IFNβ and the indicated doses of cisplatin for 72 h. For IFNβ-cisplatin sequential treatment protocol (**E**), HeLa cells were treated with 100 ng/mL IFNβ for 24, 48, or 72 h, and then cells were replated in 96-well plates and treated with the indicated doses of cisplatin for 72 h. Cell viability was examined by MTT assay. For the cisplatin-IFNβ sequential treatment protocol (**F**), HeLa cells were treated with 0.25 μg/mL cisplatin for 24 h, and then cells were replated in 96-well plates and treated with the indicated doses of IFNβ for 72 h.

The activation of intrinsic type I IFN responses in cancer cells has become a hallmark of ICD [2]. A previous study showed that type I, but not type II, IFNs contribute to chemotherapy-induced ICD, and exogenous supplementation with type I IFNs (co-administration of IFNα and IFNβ), but not type II (IFNγ), provokes the potential of cisplatin to induce ICD [20]. In this study, we accidentally found that exogenous supplementation with IFNβ by the sequential treatment protocol was sufficient to enhance the ability of cisplatin to induce the expression of ICD biomarkers. As shown in Figure 1A,B, HeLa cells were treated with the combination of IFNβ and cisplatin, either concurrently or sequentially, and then translocation of intracellular calreticulin (endo-CRT) to the plasma membrane surface (ecto-CRT, an ICD indicator [6]) was examined by flow cytometry. Although the statistical analysis suggested that IFNβ and/or cisplatin significantly induced ecto-CRT in the cotreatment group (Figure 1B), we thought that the levels of ecto-CRT might not efficiently induce ICD based on the ecto-CRT staining (Figure 1A). On the other hand, IFNβ and/or cisplatin obviously induced ecto-CRT staining (Figure 1A). The ability of sequential combination of IFNβ and cisplatin to induce ecto-CRT (1.68 ± 0.05 fold) was higher than that of concurrent combination (1.16 ± 0.05 fold), or that of IFNβ (1.49 ± 0.09 fold) or cisplatin (1.39 ± 0.04 fold) mono-treatment. In addition, the effect of sequential IFNβ (100 ng/mL) and cisplatin (2 μg/mL) treatment on ecto-CRT induction was comparable with that of oxaliplatin at the concentration of 24 μg/mL (Figure S2). In addition, sequential combination of IFNβ and cisplatin or mono-treatments did not alter the overall expression of CRT (Figure 1C), suggesting that the induction of ecto-CRT was not due to the increase of protein expression. ER stress, as indicated by phosphorylation of eIF2α, is essential for ecto-CRT and serves as a hallmark of ICD [34,35]. We found that sequential combination of IFNβ and cisplatin efficiently induced eIF2α phosphorylation compared to IFNβ or cisplatin mono-treatment (Figure 1C). Therefore, sequential, but not concurrent, treatment of IFNβ with cisplatin induced the expression of ICD biomarkers in HeLa cells. We thought that further investigation of such phenomena may provide an opportunity to identify potential molecular determinant(s) for ICD that could be activated by IFNβ pretreatment.

To exclude the possibility that different treatment protocols induced different levels of cell death, cell viability assays were performed. As shown in Figure 1D,E (the left plot), sequential combination of IFNβ (24 h treatment) and cisplatin (72 h treatment) reduced cell viability in a similar fashion as concurrent combination. This result supports the idea that the differential effects of treatment protocols on ICD biomarker expression (24 h treatment of cisplatin) are not the result of the different levels of cell viability inhibition. Interestingly, sequential treatment with IFNβ had a time-dependent trend to enhance the cisplatin-induced cell viability inhibition in HeLa cells when the treatment duration of IFNβ increased (Figure 1E). The enhancement was significantly (*p* < 0.01) observed when cells were treated first with IFNβ for 72 h (Figure 1E). In contrast, posttreatment with IFNβ did not alter the ability of cisplatin to inhibit cell viability (Figure 1F). These results indicate the therapeutic benefit of sequential IFNβ-cisplatin treatment for cancer therapy by enhancing the cytostatic/cytotoxic and immunogenic effects of cisplatin.

**Figure 2:**
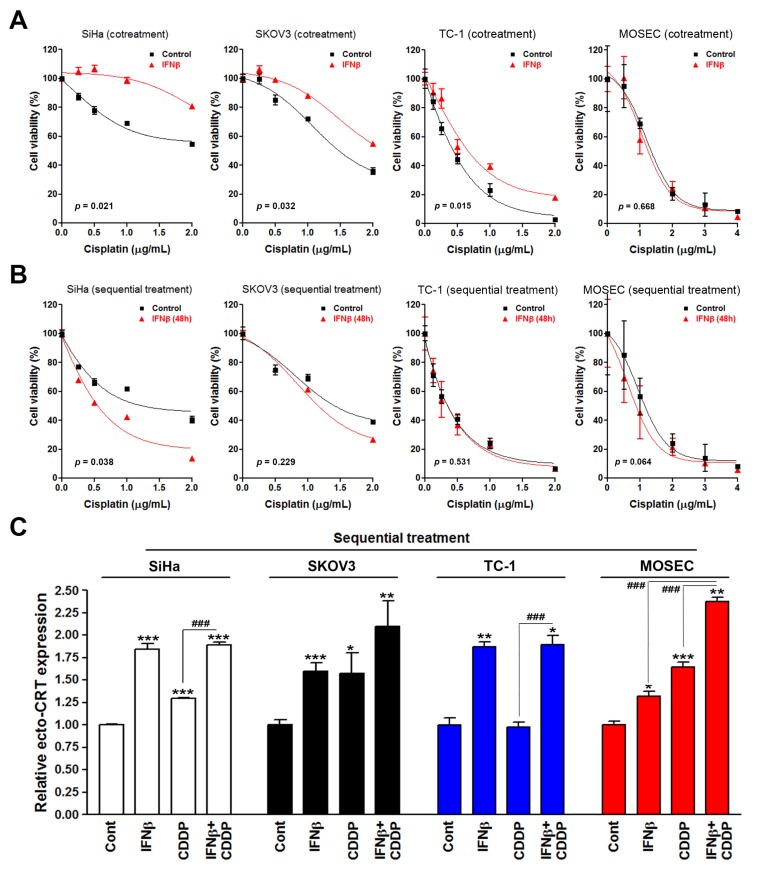
Effects of interferon β (IFNβ) and cisplatin treatment protocols on cell viability and surface calreticulin expression in cancer cells. (**A**) SiHa, SKOV3, TC-1, and MOSEC cells were cotreated with 100 ng/mL IFNβ and the indicated doses of cisplatin for 72 h. Cell viability was examined by MTT assay. (**B**) SiHa, SKOV3, TC-1, and MOSEC cells were treated with 100 ng/mL IFNβ for 48 h, and then cells were replated in 96-well plates and treated with the indicated doses of cisplatin for 72 h. Cell viability was examined by MTT assay. (**C**) SiHa, SKOV3, TC-1, and MOSEC cells were sequentially treated with 100 ng/mL IFNβ for 24 h and 2 μg/mL cisplatin for another 24 h. Surface CRT (ecto-CRT) staining was performed and analyzed by flow cytometry. The mean fluorescence of ecto-CRT was quantified and plotted. *p* < 0.05 (*), *p* < 0.01 (**) and *p* < 0.001 (*** or ^###^) indicate significant differences compared to control samples or the indicated group.

To confirm the above observations, several human and murine cervical and ovarian cancer cells were treated with the combination of IFNβ and cisplatin, either concurrently or sequentially, and cell viability assay was performed. Interestingly, we found that cotreatment with IFNβ attenuated the effect of cisplatin on cell viability inhibition in SiHa, SKOV3, and TC-1 cells (Figure 2A). In contrast, 48 h treatment with IFNβ showed a trend of enhancing the effect of cisplatin on cell viability inhibition in SiHa and SKOV3 cells (Figure 2B). Although cell type-specific effect may exist, these results support the general benefit of sequential IFNβ-cisplatin treatment for cancer therapy. The effect of sequential IFNβ-cisplatin treatment on ecto-CRT expression was also investigated. Similar to the results in HeLa cells, sequential combination with IFNβ could provoke the ability of cisplatin to induce ecto-CRT expression in SKOV3 and MOSEC cells (Figure 2C). Interestingly, IFNβ alone was sufficient to induce prominent ecto-CRT expression in SiHa, SKOV3, and TC-1 cells (Figure 2C), suggesting the essential role of IFNβ signaling in ICD induction. Taken together, sequential combination of IFNβ and cisplatin is a better way to provoke the potential of cisplatin to induce surface CRT exposure, which is irrelevant to the extent of induced cell death.

### 3.2. Gene Set Enrichment Analysis (GSEA) Identifies Potential Molecular Determinants for Oxaliplatin-Induced Immunogenic Cell Death (ICD)

**Figure 3:**
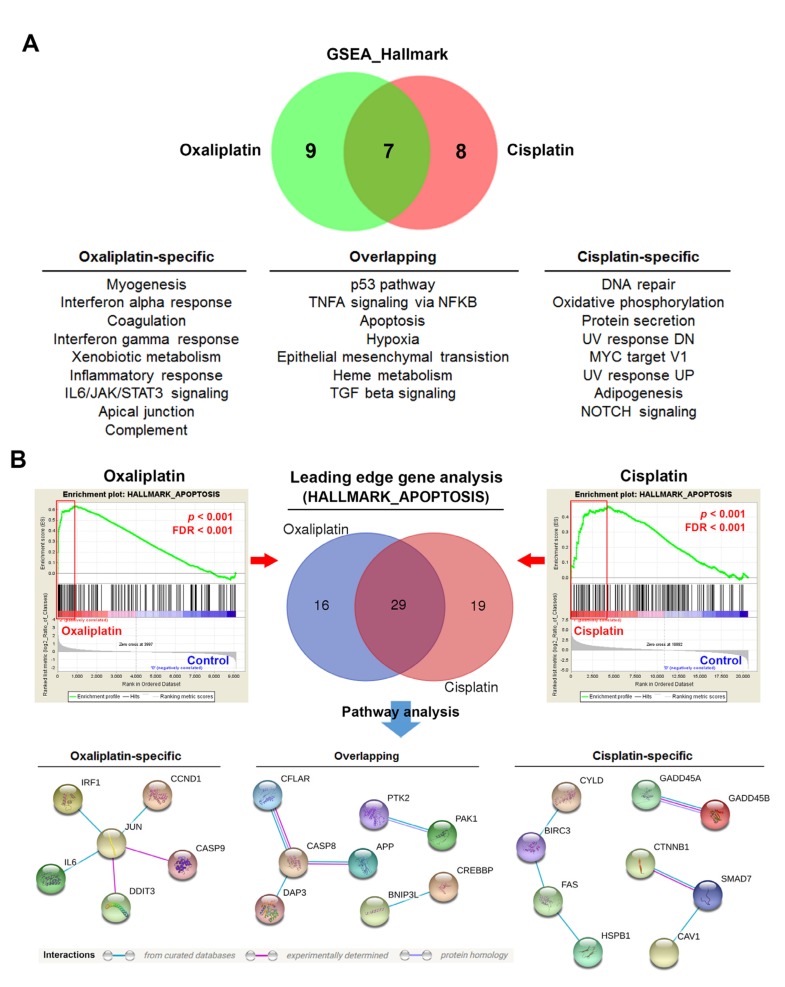
Gene set enrichment analysis (GSEA) for cisplatin- and oxaliplatin-treated A2870 cells. (**A**) The microarray data set for oxaliplatin- and cisplatin-treated A2780 human ovarian cancer cells (GSE8057) was obtained from the National Center for Biotechnology Information (NCBI) Gene Expression Omnibus (GEO) database. GSEA was performed for the enrichment of 50 cancer hallmarks. The enriched hallmarks with *p* < 0.01 and false discovery rate (FDR) with *p* < 0.25 are shown in the Venn diagram. (**B**) The enrichment plots for the apoptosis hallmark in oxaliplatin- and cisplatin-treated A2780 cells. The leading edge genes are highlighted in the red squares. The overlapping, oxaliplatin-specific, and cisplatin-specific genes were analyzed using the STRING database for network construction.

The above results indicated that treatment first with IFNβ may induce molecular signaling to provoke the potential of cisplatin to induce the surface CRT exposure. To investigate the potential molecular determinants of chemotherapy-induced ICD, a bioinformatics strategy was employed to compare the differences of cisplatin and oxaliplatin in their gene signatures. The microarray data sets for cisplatin- and oxaliplatin-treated A2780 human ovarian cancer cells (GSE8057 [26]) were obtained from the NCBI GEO database. GSEA was performed for the enrichment of 50 cancer hallmarks. The common and specific cancer hallmarks for cisplatin and oxaliplatin are shown in a Venn diagram (Figure 3A). We found that the type I IFN hallmark (interferon alpha response) was indeed specifically enriched in oxaliplatin-treated cells. To confirm the above analysis, the gene expression profile of another ICD inducer, doxorubicin, was analyzed. The microarray data sets for cisplatin- and doxorubicin-treated HeLa human cervical cancer cells (GSE72905 [27] and GSE30988 [28]) were obtained from the NCBI GEO database. GSEA was performed for the enrichment of cancer hallmarks. Because there was no enrichment in cisplatin-treated HeLa cells, the top five hallmarks are shown in Table 1 to compare the differences between cisplatin and doxorubicin. We found that the type I IFN hallmark was also enriched in doxorubicin-treated, but not cisplatin-treated, HeLa cells.

**Figure 4:**
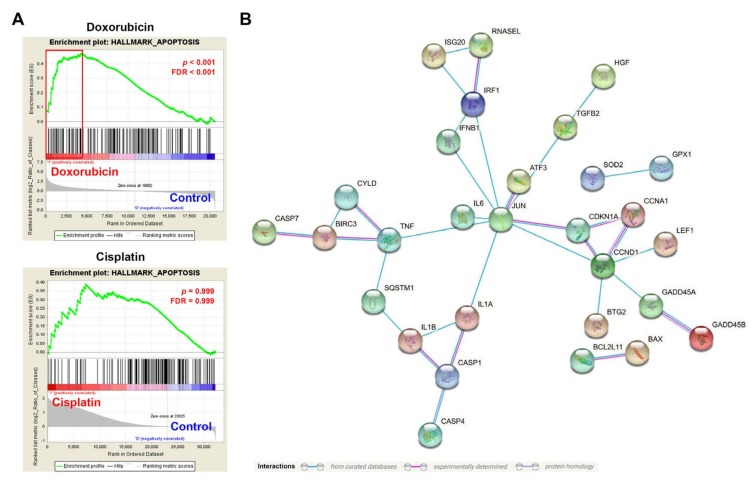
Gene set enrichment analysis (GSEA) for doxorubicin- and cisplatin-treated HeLa cells. (**A**) The enrichment plots for the apoptosis hallmark in doxorubicin- and cisplatin-treated HeLa cells. The leading edge genes are highlighted in the red squares. (**B**) The leading edge genes in doxorubicin-treated HeLa cells were analyzed using the STRING database for network construction.

Because apoptotic cancer cells by a given therapeutic drug cannot always induce ICD, we thought that efficient and inefficient ICD inducers may alter different apoptotic genes. Therefore, we analyzed their leading edge genes of the apoptosis hallmark to compare the differences of apoptosis-related genes in cisplatin- and oxaliplatin-treated cells. Then, the oxaliplatin-specific, cisplatin-specific, and common genes were subjected to network construction using the STRING database [36]. We found that oxaliplatin-specific genes can form a network by a node gene JUN (Figure 3B). Among these genes, IRF1 is related to type I IFN signaling [37,38]. We also compared the differences of apoptosis-related genes in cisplatin- and doxorubicin-treated HeLa cells. As shown in Figure 4A, the apoptosis hallmark was only enriched in doxorubicin-treated cells. Similarly, the leading edge genes can be grouped into four arms, which were connected by a node gene JUN (Figure 4B). The four arms included genes related to IFNβ signaling (IFNB1, IRF1, ISG20, and RNASEL), unfolded protein response (UPR) and apoptosis (ATF3, DDIT3/GADD153, BCL2L11/BIM, BAX, and PMAP1/NOXA), cell cycle (CDKN1A, CCND1, CCNA1, GADD45A, GADD45B, BTG2, and LEF1), and inflammasomes (IL1A, IL1B, CASP1, CASP4, SQSTM1, and CYLD). Therefore, we hypothesized that JUN-type I IFN-IRF1 signaling may play an essential role in chemotherapy-induced ICD.

### 3.3. Interferon Regulatory Factor 1 (IRF1) Is Required for the Surface Calreticulin (CRT) Exposure Induced by Sequential Interferon β (IFNβ) and Cisplatin Treatment

**Figure 5:**
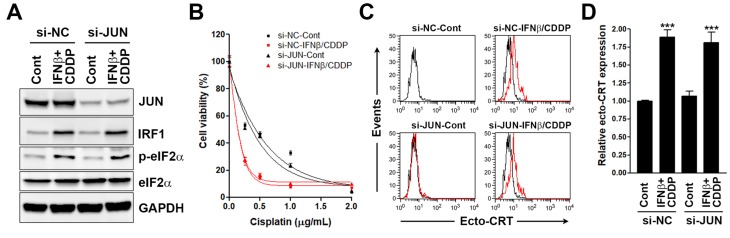
Effects of JUN knockdown on sequential interferon β (IFNβ)-cisplatin treatment-induced cell viability inhibition and immunogenic cell death (ICD) biomarker expression in HeLa cells. (**A**) HeLa cells were transfected with JUN siRNA (si-JUN) or the non-targeting control siRNA (si-NC) for 24 h, and then sequentially treated with 100 ng/mL IFNβ for 24 h and 2 μg/mL cisplatin for another 4 h. Protein expressions were analyzed by Western blotting. (**B**) JUN siRNA-transfected HeLa cells were treated with 100 ng/mL IFNβ for 48 h, and then cells were replated in 96-well plates and treated with the indicated doses of cisplatin for 72 h. Cell viability was examined by MTT assay. (**C**) JUN siRNA-transfected HeLa cells were sequentially treated with 100 ng/mL IFNβ for 24 h and 2 μg/mL cisplatin for another 24 h. Surface CRT (ecto-CRT) staining was performed and analyzed by flow cytometry. The mean fluorescence intensity in each treatment (the red line) was compared with that in untreated si-NC-transfected HeLa cells (the black line). (**D**) The mean fluorescence of ecto-CRT in (**C**) was quantified and plotted. *p* < 0.001 (***) indicates significant differences compared to control samples.

**Figure 6:**
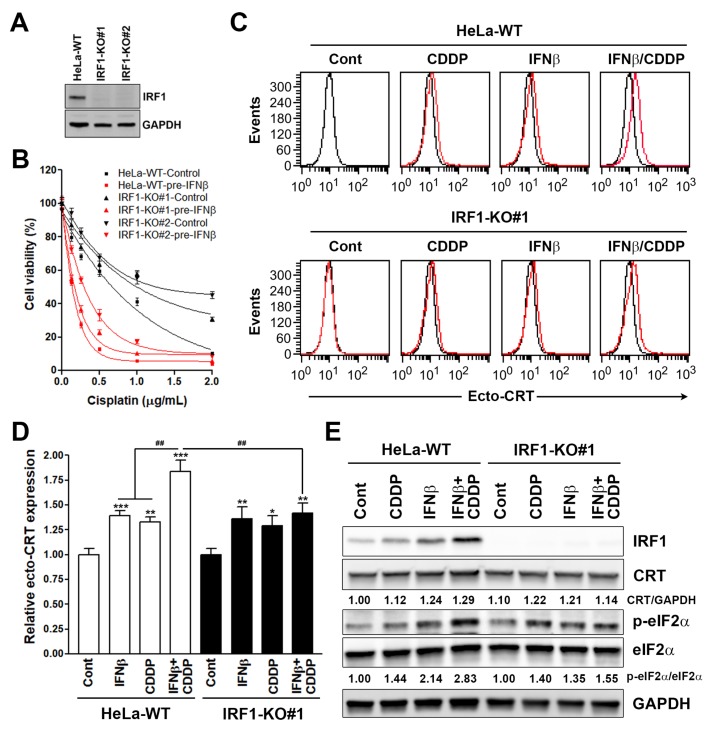
Effects of interferon regulatory factor 1 (IRF1) knockout on sequential interferon β (IFNβ)-cisplatin treatment-induced cell viability inhibition and immunogenic cell death (ICD) biomarker expression in HeLa cells. (**A**) The IRF1 expression in IRF1-knockout (IRF1-KO#1 and IRF1-KO#2) and parental wildtype (WT) HeLa cells was analyzed by Western blotting. (**B**) IRF1-knockout (IRF1-KO#1 and IRF1-KO#2) and parental (WT) HeLa cells were treated with 100 ng/mL IFNβ for 48 h, and then cells were replated in 96-well plates and treated with the indicated doses of cisplatin for 72 h. Cell viability was examined by MTT assay. (**C**) IRF1-knockout (IRF1-KO#1) and parental (WT) HeLa cells were sequentially treated with 100 ng/mL IFNβ for 24 h and 2 μg/mL cisplatin for another 24 h. Surface CRT (ecto-CRT) staining was performed and analyzed by flow cytometry. The mean fluorescence intensity in each treatment (the red line) was compared with that in untreated parental HeLa cells (the black line). (**D**) The mean fluorescence of ecto-CRT in (**C**) was quantified and plotted. *p* < 0.05 (*), *p* < 0.01 (** or ^##^) and *p* < 0.001 (***) indicate significant differences compared to control samples or the indicated group. (**E**) IRF1-knockout (IRF1-KO#1) and parental (WT) HeLa cells were sequentially treated with 100 ng/mL IFNβ for 24 h and 2 μg/mL cisplatin for another 4 h. Protein expressions were analyzed by Western blotting.

According to the above bioinformatics analyses, we first investigated the role of JUN in sequential IFNβ-cisplatin-induced growth inhibition, surface CRT exposure, and eIF2α phosphorylation by knocking down its expression using siRNA (Figure 5A). We found that sequential IFNβ-cisplatin treatment did not induce JUN expression. In addition, knockdown of JUN did not affect sequential IFNβ-cisplatin-induced IRF1 expression and eIF2α phosphorylation (Figure 5A), the cell viability inhibition (Figure 5B), or ecto-CRT expression (Figure 5C,D). We then investigated the role of IRF1 in sequential IFNβ-cisplatin-induced growth inhibition, surface CRT exposure, and eIF2α phosphorylation using the IRF1-knockout HeLa cells (Figure 6A). As shown in Figure 6B, knockout of IRF1 attenuated the cell viability inhibition by cisplatin with or without IFNβ pretreatment. Moreover, sequential IFNβ-cisplatin-induced ecto-CRT expression and eIF2α phosphorylation were attenuated by IRF1 knockout (Figure 6C–E and Figure S3). Therefore, upregulation of IRF1 by IFNβ was required for sequential IFNβ-cisplatin-induced surface CRT exposure. Interestingly, oxaliplatin-induced surface CRT exposure was also attenuated in IRF1-knockout HeLa cells (Figure S2), supporting the essential role of IRF1 in ICD.

## 4. Discussion

It is currently accepted that the efficacies of conventional chemotherapeutic agents depend on both the direct cytostatic/cytotoxic effects and indirect immuno-modulating activities including ICD induction [1]. However, ICD can only be induced by limited chemotherapeutic agents, which cannot be predicted by their structural and functional similarities [1,4,5,9]. For example, both oxaliplatin and cisplatin induce cancer cell death, partly by forming inter- and intra-strand DNA adducts. However, only oxaliplatin can efficiently trigger bona fide ICD [17,18,39]. Interestingly, accumulating evidence demonstrates that the non- and relatively weak ICD inducers can be converted into bona fide ICD inducers by combinatorial strategies [17,20,21,22], which boost their therapeutic values. In this study, we identify that pretreatment of cancer cells with IFNβ will make them more immunogenic when exposed to cisplatin later. Such sequential IFNβ-cisplatin treatment-enhanced surface CRT exposure is dependent on IRF1 expression. Thus, our study provides a novel therapeutic anticancer strategy using IFNβ as an adjuvant.

Type I IFNs are polypeptides that can activate intracellular antimicrobial programs and influence the development of innate and adaptive immune responses [38]. The most well-defined type I IFNs are IFNα and IFNβ. Most cell types produce IFNβ, whereas hematopoietic cells are the major producers of IFNα. Canonical type I IFN signaling activates the Janus kinase (JAK)-signal transducer and activator of transcription (STAT) pathway, leading to transcription of IFN-stimulated genes (ISGs) [38]. STATs can cooperate with other transcription factors to regulate target gene expression. The most established STAT-interacting transcription factors belong to the members of the IFN-regulatory factor (IRF) family such as IRF1, IRF7, IRF8, and IRF9. Interestingly, IRFs can be induced by type I IFNs through STAT-dependent pathways [38]. In contrast, IRFs play essential roles in regulating the induction of IFNα/β gene expression [37]. These findings suggest that a positive feedback regulation loop of IFNα/β and IRFs may exist. Recently, type I IFN signaling has been demonstrated as a requirement for doxorubicin-induced ICD [20]. This study found that doxorubicin stimulates the rapid production of type I IFNs by tumor cells, which is dependent on the endosomal pattern recognition receptor TLR3. By binding to IFNα and IFNβ receptors (IFNARs) on tumor cells, type I IFNs trigger autocrine and paracrine circuitries to promote the release of chemokine (C-X-C motif) ligand 10 (CXCL10). Interestingly, this study also found that both type I and type II (IFNγ) IFNs are required for doxorubicin-induced anticancer immune responses. However, type II IFNs do not participate in doxorubicin-induced ICD because of their delayed induction (5 days after chemotherapy) compared to type II IFNs (1~4 days after chemotherapy).

Recombinant IFNβs, including IFNβ-1a (Avonex, Rebif, and Plegridy) and IFNβ-1b (Betaseron and Extavia), have been approved for treating multiple sclerosis [40,41]. Several clinical trials have shown the promising results for the application of IFNβ in cancer therapy. For example, a single-institution matched case-control study in Japan demonstrated that adjuvant therapy with low-dose administration of IFNβ was beneficial for maintenance therapy in stage II and III melanoma patients without substantial toxic effects [42]. A multicenter phase I trial showed that combination therapy with IFNβ and temozolomide was safe and well tolerated and prolonged the patients’ survival in high-grade gliomas [43]. A phase II trial indicated that IFNβ given after conventional radiation therapy was well tolerated and had a survival benefit in glioblastoma [44]. However, there were also two independent phase II trials that suggested that IFNβ had limited clinical efficacy in metastatic melanoma [45,46]. Therefore, the therapeutic applications of recombinant IFNβ relies on further clinical investigations.

IRF1 has been considered a tumor suppressor [47,48,49,50,51]. IRF was identified as a STAT3-inducible proapoptotic factor that mediated chemosensitization of cervical cancer cells by the pretreatment of a pleiotropic cytokine, oncostatin M [52]. In addition, IRF1 expression is associated with response to radio/chemotherapy in cervical cancer patients [52]. Similar to this study, we also found that sequential IFNβ-cisplatin treatment-induced IRF1 expression was responsible for the enhanced anticancer activity of cisplatin. Therefore, pre-therapeutic IRF1 expression can be used as a novel predictive biomarker for chemotherapy responses, and pre-stimulation of IRF1 by cytokine therapy can enhance the efficacy of chemotherapeutic agents. It is controversial that IRF1 expression is upregulated by cisplatin, but limits drug effectiveness in ovarian cancer cells [53]. Mechanistically, cisplatin induces IRF1-dependent and p53-independent p21 expression and cell cycle arrest. Because cisplatin is more effective in proliferating cancer cells, the induction of cell cycle arrest may counteract with the anticancer activity of cisplatin [53]. However, this study does not defy the tumor-suppressive role of IRF1 because IRF1 overexpression still inhibits the transformed phenotypes of ovarian cancer cells [53]. Moreover, higher IRF1 expression is associated with better progression-free and overall survival in patients with high-grade serous ovarian carcinoma [54]. Possibly, other factors, such as p21, may also be considered with IRF1 expression for the effectiveness of chemotherapy.

Our results showed that IRF1 upregulation contributed to the phosphorylation of eIF2α by IFNβ and IFNβ-cisplatin. However, the linkage between IRF1 and eIF2α phosphorylation is still unsolved. The eIF2α can be phosphorylated by a family of four kinases, including protein kinase double-stranded RNA-dependent (PKR), PKR-like ER kinase (PERK), general control non-derepressible-2 (GCN2), and heme-regulated inhibitor (HRI) [55]. Among them, PKR, PERK, and GCN2 have been shown to be required for surface CRT exposure in ICD [17,34,56]. It has been reported that the activation of IRF1 induces PKR expression, which is partially required for IRF1-induced growth inhibition [57]. Therefore, PKR may serve as a mediator for IRF1-dependent eIF2α phosphorylation and surface CRT exposure in ICD.

ERp57, also known as protein disulfide-isomerase A3 (PDIA3) or glucose-regulated protein, 58 kD (GRP58), is an ER-localized disulfide isomerase. Although surface ERp57 itself is not immunogenic, it acts as a co-translocation partner of CRT and controls CRT surface exposure [14,58]. ERp57 can also interact with STAT3 and inhibits STAT3 signaling, which is enhanced by the ERp57-calreticulin complex formation [59]. Interestingly, inhibition of STAT3 was shown to enhance ICD [60,61,62], accounting for another way to regulate ICD by ERp57. Given the fact that IRF1 is a STAT3-inducible mediator for cell death enhancement in cervical cancer cells [57], it will be interesting to investigate the role of STAT3 and ERp57 in IRF1-depedent surface CRT exposure in the future.

In conclusion, we found that ICD inducers activate IFNβ-IRF1 signaling, leading to the surface exposure of CRT in cancer cells. Supplementation of exogenous IFNβ will upregulate IRF1 expression, which may be required for the conversion of non- and relatively weak ICD inducers into bona fide ICD inducers. Therefore, IRF1 may act as a molecular determinant for ICD induction. However, it should be noted that there are limitations to this study. For example, only two ICD biomarkers (eIF2α phosphorylation and surface CRT exposure) were used. An evaluation of other biomarkers, such as extracellular ATP and HMGB1 [6], would help to support the conclusion. Most importantly, vaccination assay, the gold-standard approach to detect ICD [6], was lacking in this study, which warrants further investigations.

## Figures and Tables

**Table 1:** The gene set enrichment analysis (GSEA) for cancer hallmarks enriched in cisplatin- and doxorubicin-treated HeLa cells.

Top 5 Hallmarks	Number of Genes in Pathway	Number of Pathway Genes Differentially Expressed (% of Total)	NES ^1^	*p* Value	FDR ^2^
Cisplatin					
Angiogenesis	36	9 (25%)	1.13	0.109	1.000
Allograft rejection	199	71 (36%)	1.04	0.210	1.000
KRAS signal DN	187	88 (47%)	1.03	0.247	1.000
Inflammatory response	197	83 (42%)	1.03	0.257	1.000
Spermatogenesis	131	56 (43%)	1.01	0.402	1.000
Doxorubicin					
Interferon alpha response	91	48 (53%)	2.72	<0.001	<0.001
Interferon beta response	193	101 (52%)	2.69	<0.001	<0.001
TFNA signaling via NFKB	198	92 (46%)	2.27	<0.001	<0.001
Inflammatory response	198	78 (39%)	2.06	<0.001	<0.001
Complement	197	68 (35%)	1.93	<0.001	<0.001

^1^ Normalized enrichment score. ^2^ False discovery rate.

## Supplementary Materials

The following are available online at https://www.mdpi.com/2218-273X/10/4/643/s1, Figure S1: Sanger sequencing of parental and IRF1-knockout HeLa cells, Figure S2: Effects of interferon regulatory factor 1 (IRF1)-knockout on oxaliplatin-induced surface calreticulin (CRT) exposure and cell death, Figure S3: Effects of interferon regulatory factor 1 (IRF1)-knockout on sequential interferon β (IFNβ)-cisplatin treatment-induced surface calreticulin (CRT) exposure and cell death.
