# Supplementary material for: Sequential Interferon β-Cisplatin Treatment Enhances the Surface Exposure of Calreticulin in Cancer Cells via an Interferon Regulatory Factor 1-Dependent Manner
Source: Biomolecules. 2020 Apr 21;10(4):643. doi: 10.3390/biom10040643 (PMC7226424; doi:10.3390/biom10040643)
Supplement: Supplementary file 1 [file biomolecules-10-00643-s001.pdf]

Supplementary Materials:

Sequential Interferon β-Cisplatin Treatment Enhances the Surface Exposure of Calreticulin in Cancer Cells via an Interferon Regulatory Factor 1-Dependent Manner

Pei-Ming Yang [1,3,4,5,6,*],Yao-Yu Hsieh [8,9], Jia-Ling Du [3], Shih-Chieh Yen [1,7] and Chien-Fu Hung [1,2,*]

[1] Department of Pathology, Johns Hopkins Medical Institutions, Baltimore, MD 21231, United States; chung2@jhmi.edu (C.-F.H)
[2] Department of Oncology, Johns Hopkins Medical Institutions, Baltimore, MD 21231, United States
[3] Graduate Institute of Cancer Biology and Drug Discovery, College of Medical Science and Technology, Taipei Medical University, Taipei 11031, Taiwan; yangpm@tmu.edu.tw (P.-M.Y.), small.lyn@gmail.com (J.-L.D.)
[4] PhD Program for Cancer Molecular Biology and Drug Discovery, College of Medical Science and Technology, Taipei Medical University and Academia Sinica, Taipei 11031, Taiwan
[5] TMU Research Center of Cancer Translational Medicine, Taipei 11031, Taiwan
[6] Cancer Center, Wan Fang Hospital, Taipei Medical University, Taipei 11696, Taiwan
[7] National Taiwan University College of Medicine, Taipei 10051, Taiwan; b00401082@ntu.edu.tw (S.-C.Y.)
[8] Division of Hematology and Oncology, Taipei Medical University Shuang Ho Hospital, 23561 New Taipei City, Taiwan; alecto39@gmail.com (Y.-Y.H)
[9] Division of Hematology and Oncology, Department of Internal Medicine, School of Medicine, College of Medicine, Taipei Medical University, 11031 Taipei, Taiwan
* Correspondence: yangpm@tmu.edu.tw; Tel.: +886-2-2736-1661 (ext.143); chung2@jhmi.edu; +1-410-502-8215

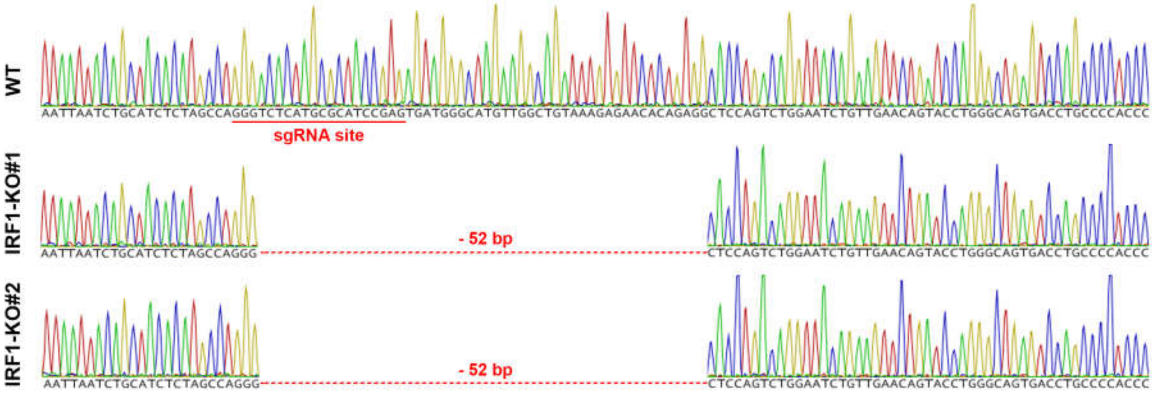

Figure S1. Sanger sequencing of parental and IRF1-knockout HeLa cells. For the validation of sgRNA target site, genomic DNA was extracted and PCR was performed using the following primer pair: GGGTGCCCTACCTCAAGAAG (forward) and AAAGAAGTCCCTCCCTTCCC (reverse). The PCR products were purified and then sequenced by the Sanger method using the forward and reverse primers.

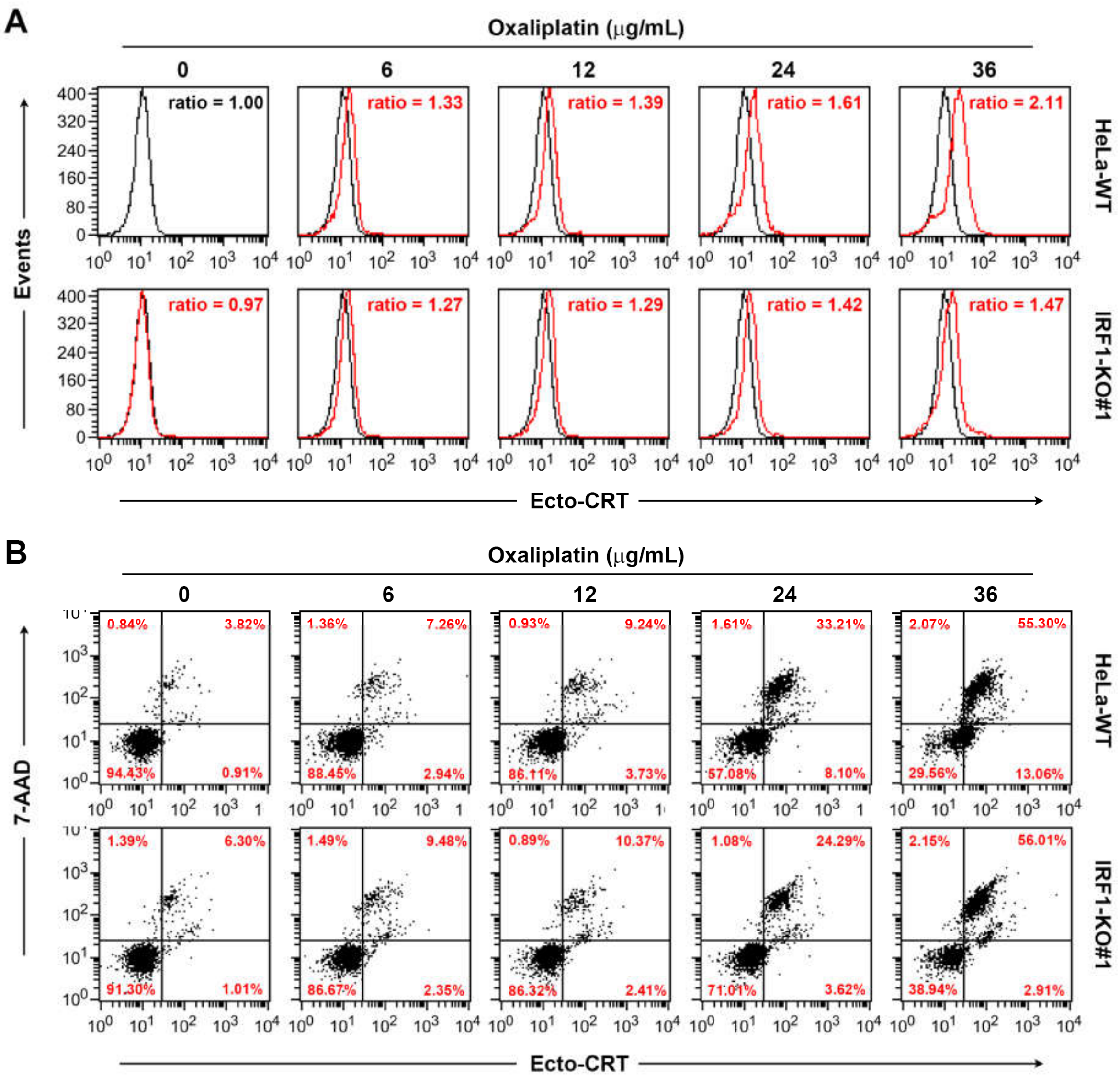

Figure S2. Effects of interferon regulatory factor 1 (IRF1) knockout on oxaliplatin-induced surface calreticulin (CRT) exposure and cell death. **(A)** Parental (WT) and IRF1-knockout (IRF1-KO#1) HeLa cells were treated with the indicated doses of oxaliplatin for 24 h. Surface CRT (ecto-CRT) staining was performed and analyzed by flow cytometry. The mean fluorescence intensity in each treatment (the red line) was compared with that in untreated parental HeLa cells (the black line), and the ratio was shown within each plot. **(B)** Parental (WT) and IRF1-knockout (IRF1-KO#1) HeLa cells were treated with the indicated doses of oxaliplatin for 24 h. Both attached and floating cells were collected for the ecto-CRT/7-AAD double staining.

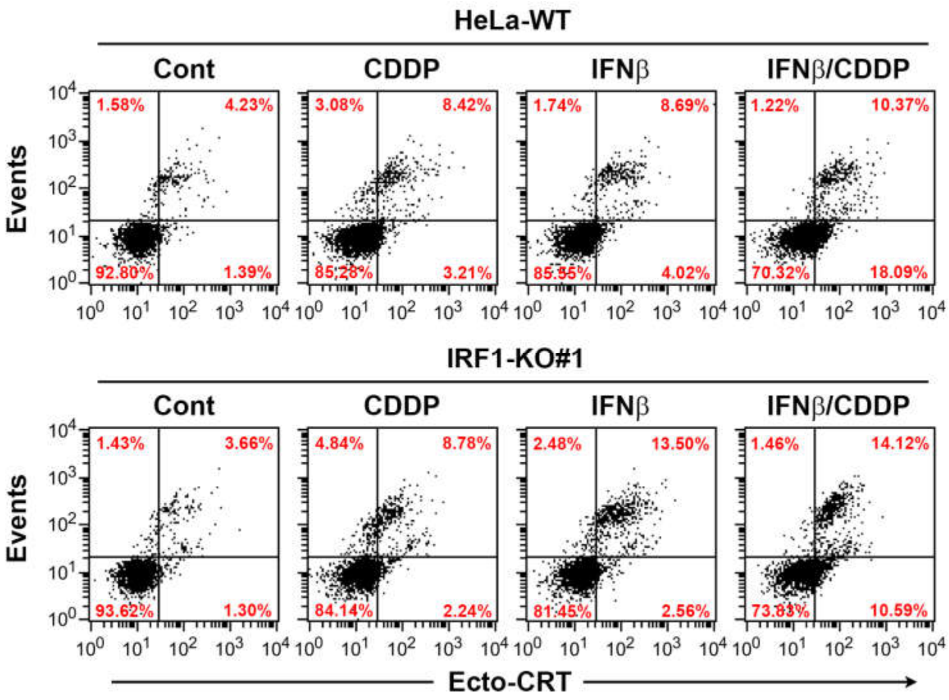

Figure S3. Effects of interferon regulatory factor 1 (IRF1) knockout on sequential interferon β (IFNβ)-cisplatin treatment-induced surface calreticulin (CRT) exposure and cell death. Parental (WT) and IRF1-knockout (IRF1-KO#1) HeLa cells were treated with 100 ng/mL IFNβ for 24 h and 2 μg/mL cisplatin for another 24 h. Both attached and floating cells were collected for the ecto-CRT/7-AAD double staining.
